# Insights from *Drosophila* on Aβ- and tau-induced mitochondrial dysfunction: mechanisms and tools

**DOI:** 10.3389/fnins.2023.1184080

**Published:** 2023-04-17

**Authors:** Vanlalrinchhani Varte, Jeremy W. Munkelwitz, Diego E. Rincon-Limas

**Affiliations:** ^1^Department of Neurology, McKnight Brain Institute, Norman Fixel Institute for Neurological Diseases, University of Florida, Gainesville, FL, United States; ^2^Department of Neuroscience, University of Florida, Gainesville, FL, United States; ^3^Genetics Institute, University of Florida, Gainesville, FL, United States

**Keywords:** Alzheimer’s disease, amyloid beta, *Drosophila*, mitochondria, neurodegeneration, tau, mitophagy, Drp1

## Abstract

Alzheimer’s disease (AD) is the most prevalent neurodegenerative dementia in older adults worldwide. Sadly, there are no disease-modifying therapies available for treatment due to the multifactorial complexity of the disease. AD is pathologically characterized by extracellular deposition of amyloid beta (Aβ) and intracellular neurofibrillary tangles composed of hyperphosphorylated tau. Increasing evidence suggest that Aβ also accumulates intracellularly, which may contribute to the pathological mitochondrial dysfunction observed in AD. According with the mitochondrial cascade hypothesis, mitochondrial dysfunction precedes clinical decline and thus targeting mitochondria may result in new therapeutic strategies. Unfortunately, the precise mechanisms connecting mitochondrial dysfunction with AD are largely unknown. In this review, we will discuss how the fruit fly *Drosophila melanogaster* is contributing to answer mechanistic questions in the field, from mitochondrial oxidative stress and calcium dysregulation to mitophagy and mitochondrial fusion and fission. In particular, we will highlight specific mitochondrial insults caused by Aβ and tau in transgenic flies and will also discuss a variety of genetic tools and sensors available to study mitochondrial biology in this flexible organism. Areas of opportunity and future directions will be also considered.

## Introduction

*Drosophila* has been used in genetic research for over 100 years (Morgan, 1910). The conservation of genes between *Drosophila* and human (Rubin et al., 2000), the simplicity of genes with lesser isoform/redundancy in flies, and the smaller size of the *Drosophila* genome compared to the human counterpart, make *Drosophila* an excellent model organism. *Drosophila* also has the advantage of having a short life cycle, around 10 days from embryo to adulthood, is easy to maintain in a small space, and is very economical. Approximately 75% of human disease-causing genes are present in the fly (Rubin et al., 2000; Reiter et al., 2001; Ugur et al., 2016) and its genome has been fully sequenced and extensively annotated (Adams et al., 2000). Fly genes can be over-expressed or knocked-down through RNAi, mutated by targeted knock-out or deletion of the gene, or replaced by knock-in of a gene. Another technical advantage in flies is the presence of the Gal4-UAS system, where the yeast transcription factor Gal4 can drive the expression of any gene of interest at any developmental stage in a tissue-specific manner (Brand and Perrimon, 1993). Thousands of Gal4 drivers and other valuable fly strains are available at stock repositories including the Bloomington *Drosophila* Stock Center (BDSC), the Harvard Transgenic RNAi Project (TRiP), the Vienna *Drosophila* Resource Center (VDRC), the Japan National Institute of Genetics (NIG) Stock Center, the Kyoto *Drosophila* Stock Center (DGGR), and the Zurich ORFeome Project (FlyORF) (Dietzl et al., 2007; Cook et al., 2010; Ni et al., 2011; Yeh et al., 2018). This is highly advantageous as researchers can easily procure essential stocks for experiments in a matter of days. Also, FlyBase is a regularly updated website that provides valuable information about any fly gene, which facilitates rapid progress in the field. All these *Drosophila* resources and tools make the fly system an ideal platform to approach the pathological complexity underlying neuronal dysfunction and degeneration.

## *Drosophila* as a model to study neurodegenerative diseases

*Drosophila melanogaster* has been used to study mechanistic aspects of human neurodegenerative diseases for over two decades (Nayak and Mishra, 2022). This is facilitated by the functional conservation of many genes and pathways between humans and flies and their remarkable similarities in neuronal functions. The nervous system of *Drosophila* is quite complex and includes functionally distinct neurons in the eyes, olfactory, gustatory and auditory organs, ventral nerve cord, brain, as well as peripheral sensory neurons (Hirth, 2010). Multiple assays have been developed to assess neurodegeneration in many of these tissues. For instance, the fly eyes contain photoreceptor neurons and are made up of 800 ommatidia that are uniformly arranged with bristles. Human disease-causing genes can be overexpressed in the eye and the effects of these genes can be determined based on the degree of alterations in eye morphology, including the size of the eye, pigmentation, ommatidial organization, and cell death. All these phenotypic outcomes are very easy to score under a dissecting microscope, making the *Drosophila* eye the preferred screening platform for modifiers of neurodegenerative processes. Apart from the eye, neurodegeneration can be also assessed based on vacuolization of the brain, locomotor performance such as climbing and flight assays, analysis of neuromuscular junction morphology, life span analysis, as well as assessment of learning and memory decline (McGurk et al., 2015; Gevedon et al., 2019). Interestingly, the accumulation of amyloid aggregates and pathological tau, the main neuropathological hallmarks in AD, can be also assessed in the fly brain by staining with Thioflavin and with antibodies against phosphorylated and conformational tau (Greeve et al., 2004). Thus, neurodegeneration-related genes and their pathways can be easily studied at molecular and pathological level in *Drosophila* to understand their impact on disease progression. In the next sections, we will discuss the contributions of *Drosophila* as experimental platform to study mitochondrial dysfunction in Alzheimer’s disease.

## Alzheimer’s disease, amyloid beta, and tau

Alzheimer’s disease (AD) is an incurable neurodegenerative brain disorder that leads to cognitive impairment and memory deficits in affected individuals (Knopman et al., 2021) and displays a reduction in the hippocampal and temporal lobe of the brain (Ramos Bernardes da Silva Filho et al., 2017). It accounts for 50–60% of dementia (Blennow et al., 2006), affecting mostly people above 65 years of age. The disease is named after the German neurologist Alois Alzheimer who examined a 51 years old patient, Auguste Deter, suffering from memory loss, hallucinations, disorientation, and language problems. Her autopsy showed an accumulation of amyloid plaques and tangles in the cerebral cortex. The etiology of the disease is not well defined. However, the amyloid cascade theory is the predominant hypothesis where cognitive deficits are due to the deposition of amyloid beta peptides forming extracellular plaques (Morishima-Kawashima and Ihara, 2002; Cummings, 2004; de Vrij et al., 2004) and subsequent hyperphosphorylation of the microtubule associated protein tau, resulting in formation of neurofibrillary tangles (NFT) (Cummings, 2004; Blennow et al., 2006; Braak et al., 2011).

A variety of amyloid beta fragments (37–43 amino acid residues) are released into the extracellular space through proteolytic processing of APP by γ-secretase (Haass et al., 2012). Among these, Abeta 42 (hereafter referred to as Aβ) is the most toxic peptide as it is highly insoluble and more prone to aggregation (Moore et al., 2018). Monomeric Aβ aggregates into oligomers and protofibrils, while insoluble amyloid beta-fibril aggregates form amyloid plaques that interfere with signaling at the synapse (Chen and Yan, 2010; Crews and Masliah, 2010). Studies have shown that Aβ aggregation causes neuronal death by altering calcium homeostasis, elevating mitochondrial oxidative stress, and reducing energy metabolism. In addition, Aβ triggers microglial priming by interacting with the microglia, making it more prone to secondary inflammations. Aβ stimulates microglia to release pro-inflammatory cytokines, and these interfere with anti-inflammatory cytokines and transforming growth factor-beta1 (TGF-β1), which can induce neuroinflammation and neurodegeneration (Torrisi et al., 2019; Merlini et al., 2021). Aβ also causes a neuroinflammatory response by activating astrocytes to release various pro-inflammatory molecules (cytokines, interleukins, complement components, nitric oxide, and other cytotoxic compounds) (Brosseron et al., 2014; van Eldik et al., 2016; Arranz and DeStrooper, 2019). Polymerization of Aβ fibrils leads its aggregation into plaques and to the activation of Glycogen Synthase Kinase 3 (GSK3), causing hyperphosphorylation of microtubule-associated Tau and subsequent formation of neurofibrillary tangles (NFT) (Eftekharzadeh et al., 2018; Tiwari et al., 2019).

Tau is a microtubule-associated protein (MAP) that plays a role in the stabilization of neuronal microtubules and regulates axonal growth. Tau exists in a set of six isoform proteins (3R0N, 3R1N, 3R2N, 4R0N, 4R1N, and 4R2N) and is expressed in neurons via alternate splicing of MAPT. Depending on the exclusion or inclusion of exon 10, the expression of tau isoforms will contain three (3R) or four (4R) microtubule-binding repeats, whereas isoforms with 0, 1, or 2 N-terminal inserts are determined by the inclusion of exons 2 and 3 (Goedert et al., 1989). The ratio of 3R to 4R is 1 in the healthy human brain (Kosik et al., 1989; Goedert and Jakes, 1990), but this ratio is altered in tauopathies (D’Souza and Schellenberg, 2005; Goedert and Jakes, 2005; Sergeant et al., 2005). Tau exists normally as soluble and unfolded protein (Mandelkow and Mandelkow, 2012) and interacts with tubulin, promoting its assembly into microtubules and helping stabilize the structure (Weingarten et al., 1975). It has the potential for multiple phosphorylation at Serine (S), Threonine (T), and Proline (P) residues within its Proline-rich region (PRG) or C-terminal region (CTR), and only 2–3 residues are phosphorylated in the healthy brain. However, there are around five to nine moles of phosphate per mole of tau in AD and other tauopathies (Grundke-Iqbal et al., 1986; Kopke et al., 1993; Holper et al., 2022). Hyperphosphorylation of Tau is associated with an aggregation of multimers and fibers (Despres et al., 2017) that seem to mediate cognitive defects. Microtubule-associated kinases such as Cyclin-dependent Kinase 5 (CDK5), Glycogen Synthase Kinase 3 (GSK3β), casein kinase II (CKII), Src-family tyrosine kinase Fyn, c-Abl tyrosine kinase (c-Abl), lemur tyrosine kinase 2 (LTK), dual specificity tyrosine-phosphorylation-regulated kinase 1A (Dyrk1A), and thousand-and-one amino acid kinases (TAOKs), casein kinase 1 (CK1), c-Jun amino-terminal kinase (JNK),extracellular signal-regulated kinases 1 and 2 (Erk1 and Erk2), adenosine-monophosphate activated protein kinase (AMPK), cyclic AMP (cAMP)-dependent protein kinase (PKA), protein kinase N1, tau-tubulin kinases 1 and 2 (TTBK1 and TTBK2), Ca2+/calmodulin-dependent protein kinase II (CaMKII) and microtubule-affinity regulating kinases (MARKs) are known enzymes to be involved in tau phosphorylation (Limorenko and Lashuel, 2022). In the AD brain, approximately 85 serine, threonine, and tyrosine residues have been found phosphorylated (Tavares et al., 2013; Limorenko and Lashuel, 2022). Therefore, deposition of extracellular Aβ plaques and intracellular accumulation of NFT constitute the main pathological hallmarks in AD (Knopman et al., 2021).

## Mitochondrial dysfunction in Alzheimer’s disease

The mitochondrion is a membrane-bound cytoplasmic organelle that carries out essential functions like ATP production through oxidative phosphorylation. It is critical for multiple cellular processes and interacts with several organelles to regulate energy metabolism (Rossmann et al., 2021; Collier et al., 2023). It is worth noting that the brain utilizes 25% of body glucose and 20% oxygen consumption, but it constitutes only 2% of body weight. As a result, the brain is very susceptible to changes in energy metabolism and, therefore, disturbances of mitochondrial functions are associated with neurodegenerative disorders (Lin and Beal, 2006; Wang et al., 2020; Zhang X. et al., 2021), including Alzheimer’s disease (Wang et al., 2014; Swerdlow, 2018). Several studies have shown that mitochondrial abnormalities are early events in AD (Reddy et al., 2012; Swerdlow, 2018). Cell lines expressing mutant APP or treated with Aβ (Schmidt et al., 2007; Diana et al., 2008; Wang et al., 2008b,^2009^; Calkins and Reddy, 2011; Manczak et al., 2011), AD mouse models (Smith et al., 1997; Li et al., 2004; Reddy et al., 2004; Caspersen et al., 2005; Manczak et al., 2006; Yao et al., 2009), and AD post-mortem brains (Parker et al., 1990; Gibson et al., 1998; Maurer et al., 2000; Butterfield et al., 2001, Dragicevic et al., 2010) are reported to have mitochondrial dysfunction. Brains from AD patients show decreased production of ATP, increased production of free radicals, lipid peroxidation, oxidative damage of DNA and protein, and cellular damage compared to control specimens (Parker et al., 1990; Gibson et al., 1998; Maurer et al., 2000; Wang et al., 2005; Devi et al., 2006; Dragicevic et al., 2010), suggesting that mitochondrial dysfunction is a main pathological feature of AD.

**FIGURE 1:**
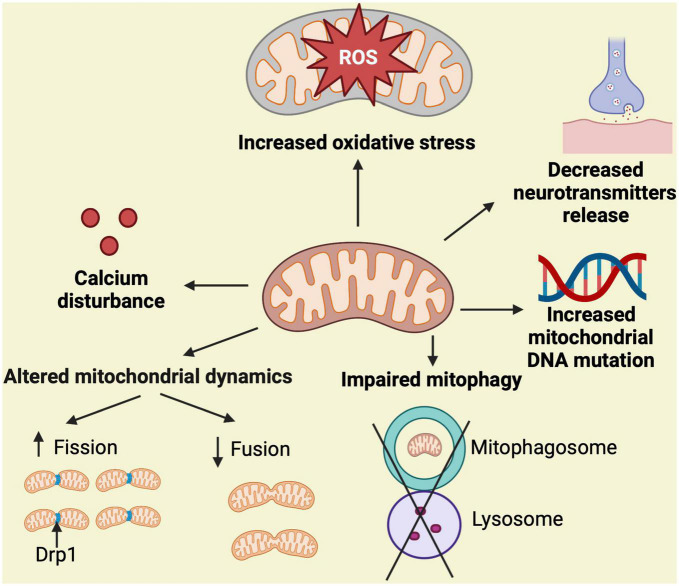
Simplified overview of mechanisms associated with mitochondrial dysfunction. Mitochondrial homeostasis is regulated by mitochondrial dynamics (fission and fusion), proper handling of calcium, control of ROS production, neurotransmitters function, maintenance of mtDNA replication, and elimination of damaged mitochondria through mitophagy. Stressful and persistent insults to the mitochondria, such as the presence of neurotoxic proteins, alter these processes resulting in the indicated abnormalities, which leads to mitochondrial dysfunction and potential cell death. Fission eliminates abnormal mitochondria from healthy ones and this process is mediated by Drp1.

The mitochondrial cascade hypothesis was first reported in 2004 (Swerdlow and Khan, 2004). According to this hypothesis, mitochondrial dysfunction disrupts multiple pathways connected to AD, resulting in a variety of clinical phenotypes including cognitive decline. It also states that mitochondrial dysfunction alters Aβ homeostasis triggering its overproduction and accumulation, and that an overall bioenergetic dysfunction might be the main culprit in AD [reviewed in Swerdlow (2023)]. Accordingly, the mitochondrial ROS production, calcium homeostasis, mitochondrial morphology and number, transport along the neuronal axon, neurotransmitters levels, mitophagy, and mtDNA mutation and oxidation, are all clearly compromised in AD (Johnson and Blum, 1970; Hirai et al., 2001; Hauptmann et al., 2009; Wang et al., 2009; Calkins and Reddy, 2011; Butterfield and Halliwell, 2019; Wong et al., 2020; Figure 1). Next, we will examine specific mechanisms mediating mitochondrial dysfunction and abnormalities in the context of AD.

## Mitochondrial ROS and oxidative stress

The mitochondrion contributes with approximately 90% of cellular reactive oxygen species (ROS), a by-product of electron transport of aerobic respiration in mitochondria (Balaban et al., 2005). When cellular homeostasis is disrupted, mitochondria produce less ATP and more ROS, resulting in oxidative stress (Gibson et al., 1998). AD patients are reported to have increased oxidative damage of proteins, nucleic acids, sugars, and lipids (Butterfield and Halliwell, 2019). For instance, markers of protein oxidation, such as protein carbonyl content, are significantly increased in the parietal lobe, superior, and middle temporal gyrus, and hippocampus of AD brains (Hensley et al., 1995; Lyras et al., 1997; Aksenov et al., 2001). Similarly, 3-nitrotyrosine, another protein oxidative modification, was significantly increased in various brain regions and cerebrospinal fluid from AD cases (Good et al., 1996; Smith et al., 1997; Tohgi et al., 1999; Castegna et al., 2003; Reed et al., 2009; Butterfield and Halliwell, 2019). Oxidative damage of DNA/RNA in AD brains causes double-strand breaks of DNA, crosslinking of DNA/DNA or DNA/protein, and extensive modification of DNA bases. Consequently, levels of DNA breaks in the AD hippocampus and cerebral cortex are found to be high (Mullaart et al., 1990; Anderson et al., 1996). Both mitochondrial DNA and nuclear DNA from AD brains have a significant increase of 8-hydroxydeoxyguanosine (8-OHdG) and 8-hydroxyguanosine (8-OHG), markers of DNA oxidation (Mecocci et al., 1994; Good et al., 1996; Lyras et al., 1997). Similarly, the levels of oxidized rRNA or mRNAs are significantly elevated in AD cases (Shan et al., 2003; Ding et al., 2005; Honda et al., 2005). Moreover, lipid peroxidation products such as 4-hydroxynonal, malondialdehyde (MDA), and 2-propenal (acrolein) are increased in different regions of AD brains (Wang et al., 2014). In contrasts, the level of antioxidant factors and enzymes is decreased in AD brains compared to control specimens (Marcus et al., 1998; Doré, 2002; Kim et al., 2006; Venkateshappa et al., 2012). Altogether, these observations suggest that mitochondrial dysfunction and oxidative stress are closely linked and, thus, are considered primary triggers of neuronal death.

## Calcium dyshomeostasis

The mitochondria play an essential role in the maintenance of calcium homeostasis in neurons which is essential for their survival (Jouaville et al., 1999). The level of mitochondrial calcium affects the activity of mitochondria and the supply of ATP. The mitochondrial calcium uniporter (MCU) protein complex conducts calcium transport into the matrix through MICU1 and MICU2/3 proteins (MICU gatekeepers) that sense calcium levels (Perocchi et al., 2010). AD patients have reduced expression of cytosolic calcium-binding proteins calmodulin, calbindin D28K, and parvalbumin which might cause activation of MCU after free calcium binds to MICU1 and MICU2/3 (McLachlan et al., 1987; Riascos et al., 2011; Ahmadian et al., 2015; Ali et al., 2019). Therefore, the level of calcium in mitochondria is elevated in AD. Consistently, exposure of cortical neurons to Aβ increases calcium concentration and promotes neurodegeneration, which is rescued by blocking MCU proteins (Hedskog et al., 2013). Aβ oligomers are also reported to form a calcium-permeable pore in the mitochondrial membrane that regulates calcium uptake and may disrupt the homeostasis of calcium in the mitochondria (Lashuel et al., 2002; Shirwany et al., 2007). In addition, Aβ oligomers promote the release of calcium stored in the ER to increase mitochondrial calcium levels, leading to mitochondrial dysfunction (Ferreira et al., 2015). Indeed, the expression of Ryanodine receptors (RyR), which regulate the release of intracellular Ca2 + in the ER, varies during AD progression and the RyR fly homologue has been found as modifier of Abeta and tau toxicity in transgenic flies (Casas-Tinto et al., 2011; Feuillette et al., 2020). Calcium homeostasis also depends on the sarcoplasmic/endoplasmic reticulum Ca2 + ATPase (SERCA), an essential ER protein that pumps Ca2 + into the ER, and modulation of SERCA expression modifies Aβ levels (Green et al., 2008). However, the direct role of RyR and SERCA in the AD-related mitochondrial malfunction remains to be elucidated. On the other side, it has been demonstrated that dyshomeostasis of mitochondrial calcium can be restored through calcium efflux pathways, mainly through the sodium-calcium exchanger (NCLX) (Boyman et al., 2013). Interestingly, AD pathology in mouse models is associated with the loss of mitochondrial NCLX expression and function, and rescue of NCLX in these mice restores both cognitive decline and cellular pathology (Jadiya et al., 2019). Additional studies in AD mouse models demonstrated that an elevated mitochondrial calcium level is associated with induction of apoptosis, highlighting the importance of mitochondrial calcium concentration in this process (Sanz-Blasco et al., 2008; Calvo-Rodriguez et al., 2020). Of note, a recent study found an oscillation of cytosolic Ca2 + in primary neurons after incubation with tau K18, a protein fragment carrying the four repeat (4R) domain of the protein. As a result, both mitochondrial and cytosolic Ca2 + are increased, indicating that Tau also impairs Ca2 + homeostasis (Britti et al., 2020).

## Mitochondrial fusion and fission

Mitochondria undergo fission and fusion in the cytoplasm to maintain proper distribution (Zhu et al., 2013), and disruption of either of these processes leads to neurological disorders (Mishra and Chan, 2014). The fission of mitochondria is regulated by the GTPase-related dynamin-related-protein1 (Drp1), also known as Dynamin-1-like protein (DLP1) in humans. It triggers the fragmentation of mitochondria and acts as a mitochondrial fission factor; however, its downregulation promotes mitochondrial fusion (Wenger et al., 2013). Of note, the structural damage in mitochondria was documented in AD brains over two decades ago (Hirai et al., 2001). Subsequent studies demonstrated that AD brains have a reduction of mitochondrial size and number (Wang et al., 2008b,^2009^), which could cause a shortage of mitochondrial bioenergetics either by enhancing ROS generation (Yu et al., 2006) or by having a negative effect on electron transport chain (ETC) function (Liu et al., 2011; Zhou et al., 2017). AD patients are reported to have increased mitochondrial fission (Manczak et al., 2011; Kandimalla et al., 2016). Interestingly, the pathology of AD in the brain not only includes an interaction between oligomeric Aβ and DLP1, but also an interaction between hyperphosphorylated tau and DLP1 (Manczak et al., 2011). This suggests that these abnormal interactions induce mitochondrial fragmentation, which is supported by *in vivo* and *in vitro* models of AD. For instance, transgenic flies expressing Aβ display abnormal dynamics and distribution of mitochondria (Iijima-Ando et al., 2009; Zhao et al., 2010). In neuronal cell culture, where Aβ or APP is overexpressed, mitochondria undergo fragmentation with altered distribution (Wang et al., 2008b,^2009^; Manczak et al., 2011). In M17 neuroblastoma cells and primary neurons, wild-type or mutant APP overexpression also induces fragmentation of mitochondria (Wang et al., 2008b,^2009^). Interestingly, APP- or Aβ-induced mitochondrial deficit in neurons is rescued by blocking mitochondrial fission, confirming the role of this process in AD pathogenesis (Wang et al., 2008b,^2009^). On the other hand, mitochondria fusion is regulated by dynamin-related GTPase proteins mitofusin-1 (Mfn1), mitofusin-2 (Mfn2), and the optic atrophy type 1 (OPA1) protein where Mfn1 and Mfn2 bind to the outer membrane of mitochondria and OPA1 binds to the inner mitochondrial membrane and mediates the fusion of the inner mitochondrial membrane (Koshiba et al., 2004; Song et al., 2009). In mice cortexes and the hippocampus, knockout of Mfn2 caused structural and functional damage to mitochondria as well as neuroinflammation, oxidative stress and neuronal death, illuminating the role of mitochondrial fragmentation in AD pathology (Jiang et al., 2018; Han et al., 2020). Confocal and electron microscopy studies from another group confirmed the structural damage and fragmentation of mitochondria in the brain of CRND8 APP transgenic mice at 3 months of age, much before visible amyloid deposition, suggesting that abnormal mitochondrial dynamics is an early event in AD pathogenesis (Wang et al., 2017). It is worth noting that overexpression of Tau also causes abnormal mitochondrial fusion (Li et al., 2016; Kandimalla et al., 2018). A study showed that overexpression of human wild-type tau alters mitochondrial dynamics and results in mitochondrial elongation by increasing the fusion proteins OPA1, Mfn1, and Mfn2, which reduces neuronal viability (Li et al., 2016; Szabo et al., 2020). In addition, the knockdown of mfn2 reduced the human tau-enhanced mitochondrial fusion and restored mitochondrial function, suggesting that Mitofusin-associated mitochondrial fusion might contribute to tau toxicity (Szabo et al., 2020). Another group has shown that expression of caspase-cleaved tau in cortical neurons from tau-/- knockout mice, as well as in immortalized cortical neurons, led to mitochondrial fragmentation with a decline in OPA1 levels (Pérez et al., 2017; Szabo et al., 2020), indicating the impact of tau on mitochondrial fusion.

## Axonal trafficking deficits and abnormal mitochondrial distribution

In addition to defects in mitochondrial structure, reduced expression of mitochondrial fission and fusion proteins in AD can lead to the absence of mitochondria in axons or dendritic segments (Wang et al., 2009; Pickett et al., 2018). Mitochondria undergo anterograde and retrograde transport that helps maintain healthy mitochondria by inhabiting axons with fresh mitochondria and recycling the damaged ones (Sheng and Cai, 2012; Lin et al., 2017). Alteration of either of these processes causes a reduction of healthy mitochondria, which leads to impaired mitochondrial function. Dysregulation of axonal mitochondrial transport contributes to AD as this transport is crucial for the maintenance of neurons and their synaptic function (Hollenbeck and Saxton, 2005). In mammals, Milton (OIP106 and GRIF1) and Miro (Miro1 and Miro2) regulate the attachment of mitochondria to microtubules via kinesin heavy chains (Fransson et al., 2006). In *Drosophila*, Milton and Miro proteins perform similar functions (Guo et al., 2005) and, thus, mitochondria are absent in synaptic terminals and axons when there is no Milton or Miro (Stowers et al., 2002). Interestingly, it has been reported that the number of mitochondria is reduced in the axons of hippocampal neurons upon Aβ treatment (Du et al., 2010), which is consistent with a lower number of mitochondria in axons from AD brains (Stokin et al., 2005). Mislocalization of mitochondria and its decrease in dendrites, axons, and soma (Iijima-Ando et al., 2009) was observed as consequence of Aβ expression in flies. Similar results were reported in neuronal cell cultures exposed to Aβ oligomers, which led to reduced mitochondrial trafficking/motility in axons (Du et al., 2010; Wang et al., 2010; Calkins and Reddy, 2011; Rui and Zheng, 2016). On the other hand, tau has been also associated with abnormal mitochondrial trafficking. For instance, earlier investigations showed that over-expression of tau in neuroblastoma cells alters the kinase-dependent anterograde axonal transport of mitochondria by enhancing the binding of microtubules, resulting in neurites almost devoid of these organelle (Ebneth et al., 1998). *In vivo* studies in transgenic mice confirmed the reduced mitochondrial movement in axons upon overexpression of human tau (Stoothoff et al., 2009; Vossel et al., 2015). Intriguingly, not only the expression of tau but also its phosphorylation impairs mitochondrial transport. For instance, in neuronal PC12 cells and mouse cortical neurons, phosphorylation of tau at AT8 sites (Ser 199, Ser202, and Thr 205) inhibits mitochondrial movement (Shahpasand et al., 2012). This is consistent with abnormal mitochondrial distribution in neurons positively stained with the anti-phospho tau Alz50 antibody in AD brains (Kopeikina et al., 2011), suggesting that tau plays a key role in the mitochondrial loss observed in AD (Wee et al., 2018).

## Neurotransmitters and mitochondrial dysfunction

Mitochondrial dysfunction affects a number of neurotransmitters in AD. For instance, memory and learning deficits result from insufficient cholinergic transmission. In this case, the hyperpermeability of the mitochondrial membrane leads to degeneration of cholinergic neurons and deficiency of acetylcholine (ACh) (Wong et al., 2020). Dysfunctional mitochondria also alter the activity of acetylcholine esterase (AChE) and recycling of choline from the synapse is hampered by mitochondrial-induced oxidative stress via nitrosative stress, which results in ACh deficiency (Wong et al., 2020). Serotoninergic, dopaminergic, norepinephrinergic, and histaminergic systems comprise the diverse monoaminergic neurotransmission network. Through membrane permeabilization and altered serotoninergic metabolism, mitochondrial dysfunction in AD causes serotoninergic inefficiency (Yamamoto and Hirano, 1985; Lai et al., 2011). Mitochondrial dysfunction also causes the loss of serotoninergic neurons via caspase-dependent apoptosis resulting in the reduction of 5-hydroxytryptamine (5-HT) or Serotonin neurotransmission (Wong et al., 2020). In AD, an excessive 5-HT breakdown is the result of mitochondrial dysfunction, which leads to a 5-HT deficit. The inadequate serotoninergic transmission also contributes to AD progression by causing ROS accumulation and further mitochondrial dysfunction (Wong et al., 2020).

## Impaired mitophagy

Mitophagy is a mechanism that eliminates damaged mitochondria by activating PINK1 at the outer mitochondrial membrane (Nguyen et al., 2016). Studies have shown that Parkin, an E3-ubiquitin ligase, is drawn to the mitochondria by PINK1 and phosphorylated there to initiate the mitophagy pathway for protein ubiquitination and degradation (Geisler et al., 2010; Matsuda et al., 2010; Narendra et al., 2010; Vives-Bauza et al., 2010). Accumulation of damaged mitochondria is due to insufficient mitophagy to remove them (Ye et al., 2015) or to defects in lysosomal degradation (Martín-Maestro et al., 2016, 2017; Kerr et al., 2017; Sorrentino et al., 2017). Nonetheless, it has been suggested that AD is mainly associated with mitophagy impairment (Nixon and Yang, 2011). For instance, post-mortem hippocampal brain samples from AD patients showed a reduction of mitophagy by 30–50% compared to control patients (Fang et al., 2019). Proteins believed to be involved in autophagy and mitophagy processes, such as Optineurin (OPTN), ATG5, ATG12, Beclin-1 (Bcl-1), PI3K class III, ULK1, AMBRA1, BNIP3, BNIP3L, FUNDC1, VDAC1, and VCP/P97 were decreased in AD brains (Martín-Maestro et al., 2017). The mitochondrial structure and function in the brains of AD patients and mouse models change into a swollen round shape with deformed cristae, low ATP production, reduced LC3 recruitment to the mitochondria, dysfunctional AMP-activated protein kinase (AMPK), and inhibition of its targets ULK1 and TBK1, which collectively impairs the mitophagy process (Hirai et al., 2001; Martín-Maestro et al., 2016; Wang et al., 2017; Fang et al., 2019). Parkin’s ability to translocate to damaged mitochondria is also affected by abnormal contacts between the projection domain of tau protein and Parkin, which prevents mitophagy (Cummins et al., 2019). Moreover, addressing mitophagy with urolithin A or actinonin improves memory in APP/PS1 mice and *C. elegans* expressing Aβ or tau, emphasizing the role of mitophagy in AD pathogenesis (Fang et al., 2019).

## Mitochondrial genome abnormalities

Mitochondria have their own DNA called mtDNA that codes for the 13 mitochondrial core proteins of the electron transport chain complexes, two rRNAs, and 22 tRNAs (Taanman, 1999; D’Souza and Minczuk, 2018). Although mitochondrial DNA plays a crucial role in mitochondrial function, it is prone to mutations due to the lack of histone proteins necessary to protect DNA and to mediate DNA repair mechanisms (Yana et al., 2013; Boczonadi et al., 2018). MtDNA mutations, via inheritance or gradual somatic mutation, affect mitochondrial function, which leads to cell death and disease (Swerdlow, 2018). Like AD patients, individuals with mtDNA mutations are reported to have similar cognitive deficits.

(Inczedy-Farkas et al., 2014), suggesting the potential role of mtDNA in cognition. In the case of AD, affected individuals have an increase in mtDNA mutations, possibly due to higher oxidative damage (Swerdlow, 2018). Indeed, AD patients have 10-fold higher levels of oxidized bases in mtDNA than nuclear DNA compared to healthy controls (Mecocci et al., 1994; Wang et al., 2005). This is consistent with higher oxidized nucleic acid in mtDNA in MCI patients and preclinical AD (Lovell et al., 2011). All these observations illustrate the role of mitochondrial genome abnormalities in AD pathogenesis.

## Assays for studying mitochondrial biology

Many assays have been developed and improved over the years to analyze the structure and function of the mitochondria. Flow cytometry is a standard assay to record mitochondrial mass (Doherty and Perl, 2017). However, it was only in recent decades that this procedure could be performed independently of mitochondrial potential due to the production of the MitoTracker family of dyes in the mid-1990s. Specifically, MitoTracker probes-green (MTG) is used for its ability to accumulate inside the mitochondria despite the measured membrane potential, unlike the positively charged red dye (Doherty and Perl, 2017; Rana et al., 2017). MTG collects inside the mitochondrial matrix and binds to free thiol groups in cysteine residues located on the mitochondrial membrane proteins; the amount of accumulated dye can then be quantified via fluorescence relative to the mitochondrial size (Presley et al., 2003; Doherty and Perl, 2017).

Along with the size, it is also important to measure the mitochondrial membrane potential (MMP) to assess the global mitochondrial function, as this parameter directly correlates with the ability of the mitochondria to generate ATP for the cells. The MMP is created from the electrochemical gradient formed through a series of coupled redox reactions in the electron transport chain steps; decreases in the MMP can be detected using lipophilic cationic fluorescent dyes (Sakamuru et al., 2012). Other membrane-potential dependent fluorescent dyes that can be used to measure MMP include tetramethylrhodamine, methyl ester (TMRM), and tetramethylrhodamine, ethyl ester (TMRE). The more polarized the mitochondria, the brighter the signal when measured due to higher accumulation of dye (Perry et al., 2011). Other dyes have been developed to analyze and measure oxygen consumption, which is directly related to oxidative phosphorylation (OXPHOS) activity (Yarosh et al., 2008). Spectrophotometric assays have also been developed to measure the activity of mitochondrial enzymes or the concentrations of cellular metabolites. For instance, by measuring enzyme activity through spectrophotometry, it was found that the activity of the mitochondrial complex I is slightly reduced in the brain during aging, but highly reduced in the context of neurodegeneration (Pollard et al., 2016).

In many cell types, the mitochondria create a complex, reticular network to support its critical functions, such as energy production. These networks undergo constant dynamic changes, which require the complementary processes of fission and fusion to occur (Hoppins et al., 2020). Several *in vitro* and *in vivo* studies were performed to assess mitochondrial fission and fusion and earlier assays provided evidence that mitochondrial fission involves multiple constriction steps that are marked by the endoplasmic reticulum (ER) and dynamin-related proteins (DRPs) (Friedman et al., 2011; Anand et al., 2014). Recent advances in light and electron microscopy demonstrated that the inner and outer membrane dynamics may be uncoupled during fission and fusion, which suggests that both processes are uniquely required to uphold the morphology of the mitochondria during its dynamic equilibrium; problems in this relationship can cause a diverse array of diseases as mitochondrial fission and fission are required for mitochondrial replication, a process critical to maintain energy levels and overall cell health (Cho and Sun, 2020; Hoppins et al., 2020). To correctly visualize mitochondrial fusion, stable cell lines that express fluorescent mitochondrial matrices via targeted proteins must be generated; once this is done, the mitochondria can be isolated, and the fusion assay can be performed (Hoppins et al., 2020). The fusion efficiency of the mitochondria can be quantified by dividing the number of fused mitochondria by the total number of mitochondria seen in the microscope field. It is anticipated that about 15–20% of total mitochondria are fused in wild-type control specimens (Hoppins et al., 2020).

Mitochondrial fission is required for growing and dividing cells and is mediated by the cytosolic dynamin family member, Drp1, in *Drosophila* and mammals (Youle and van der Bliek, 2012). Assays to measure fission *in vitro* involve quantification of the GTPase cycle kinetics and biochemical activity of Drp1 (Ingerman et al., 2005). Electron microscopy has also been included to study mitochondrial fission through Drp1-mediated liposome tubulation and constriction assays. Once images are obtained, the ultrastructural changes can be analyzed and measured using the ImageJ software (Schneider et al., 2012).

## Mitochondrial biology in *Drosophila*

*Drosophila* is a prominent model for studying mitochondrial diseases because researchers can manipulate the mitochondrial genome to express the characteristics of many human mitochondrial disorders (Chen et al., 2019). By utilizing certain restriction enzymes on *Drosophila* mitochondrial DNA, it has been observed that many mitochondrial mutations are heritable by isolation of specific disease-causing genes; when these methods are used with genetic drivers, such as GMR-GAL4, heteroplasmic flies with different mitochondrial genomes can be produced in order to study the molecular and phenotypic effects of a specific disease gene (Xu et al., 2008; Chen et al., 2015).

From a genomics perspective, human and *Drosophila* genomic mtDNA are very similar despite the human genome being about 3 kb shorter (Lewis et al., 1995). The 16,559 kb human mtDNA genome encodes for 13 proteins, 22 tRNAs, and two rRNA; all thirteen of these mitochondrial proteins make up components of the four complexes found in the electron transport chain, and almost all the DNA sequences in each specific protein have small introns that must be spliced out before translation can occur. *Drosophila* mtDNA encodes the same transcripts as its human counterpart but has a slightly different genomic order, predominantly due to its expanded “A + T-rich” regions (Sen and Cox, 2017). Crucial molecular functions of the mitochondria, including transport, oxidative phosphorylation (OXPHOS), and nucleotide biosynthesis, have highly conserved nuclear-encoded genes across both species, making *Drosophila* an excellent model for studying metabolic issues (Chen et al., 2019). Each of the 13 mitochondrial proteins is translated in the mitochondrial matrix using mtDNA-encoded tRNAs and most of the mRNA sequences for the mitochondrial proteins are separated by at least one mtDNA-encoded tRNA. It is critical that each mtDNA-encoded tRNA is properly and systematically excised since both human and *Drosophila* mtDNA are transcribed in a polycistronic manner. Without proper excision of each previous transcript, normal translation and processing of the proteins cannot occur which can lead to illnesses or even the death of the organism (Sen and Cox, 2017).

*Drosophila* research can also be used to define phenotypes associated with defective mitochondria. It has become clear that the number of mitochondria present in different organisms can fluctuate and the structure of the organelle dynamically changes depending on which type of cell they reside in Zhang et al. (2016). Neuronal cells are in a constant state of high energy demand so they are considered to be especially vulnerable to dysfunction in the mitochondrial equilibrium as this can lead to abnormally low levels of ATP; specifically, there is an abundance of mutations in the MFN2 gene that have been commonly linked to a peripheral neuropathy caused by Charcot-Marie-Tooth disease Type 2A (CMT2A) (Kyriakoudi et al., 2021). Severe symptoms of this disease, most likely caused by mutations in the functional domains of the gene, include distal limb atrophy leading to loss of leg function as well as distal sensory loss in the limbs (Züchner et al., 2004). Other mutations in the *MFN2* gene can be linked to other CMT diseases, which can damage the optic nerve and other crucial neurons in different sensory pathways (Zhou et al., 2019). In addition to genetic research, behavioral assays such as flight and climbing tests can be performed in flies as behavioral defects correlate with key features observed in patients with certain mitochondrial disorders (Jacobs et al., 2004).

## Tools for studying mitochondrial biology in flies

**FIGURE 2:**
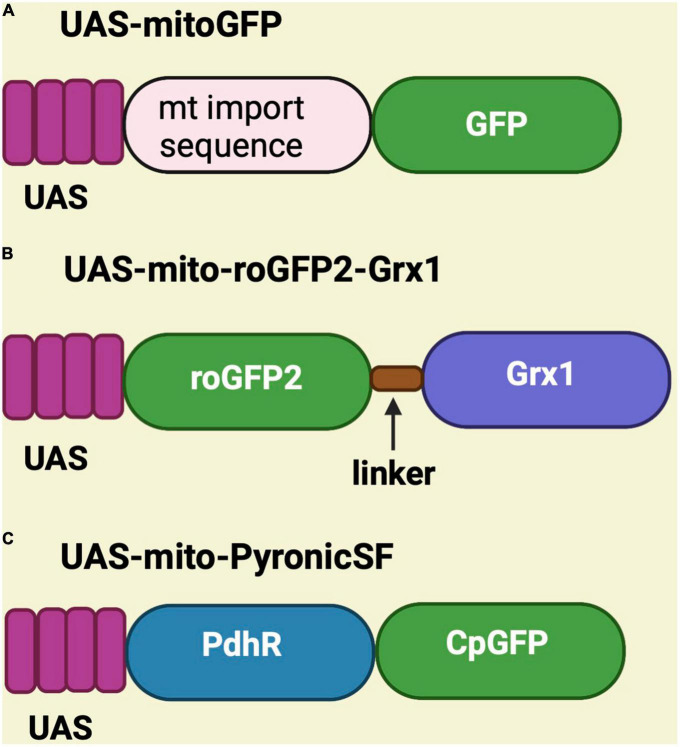
Schematic representation of UAS-mitoGFP, UAS-mito-roGFP2-Grx1 and UAS-mito-PyronicSF constructs. **(A)** UAS-mitoGFP contains a mitochondrial import sequence fused to GFP and serves as mitochondrial marker. **(B)** UAS-mito-roGFP2-Grx1 is a redox sensor transgene in which glutaredoxin-1 (Grx1) is fused to redox-sensitive GFP. **(C)** UAS-mito-PyronicSF is a pyruvate sensor containing the bacterial pyruvate-sensitive transcription factor PdhR linked to circulated permuted GFP (CpGFP), which allows real-time assessment of mitochondrial pyruvate transport.

Genetically encoded sensors have been generated in *Drosophila* to monitor mitochondrial structure, function, and metabolites. These sensors are placed under the control of the UAS promoter and have been used to perform mitochondrial assessments in different fly tissues using tissue-specific Gal4 drivers such as C155-Gal4 (pan-neuronal), MB-GS Gal4 (mushroom body), MB296B-Gal4 (dopaminergic neurons), TH-Gal4 (dopaminergic neurons), vGlut^*OK*371^ Gal4 (glutamatergic neurons), esg*^ts^*-Gal4 (intestinal stem cell), apt-Gal4 (perineurial glial cells), and vGlut^*VGN*6341^ Gal4 (glutamatergic interneurons) (Hwang et al., 2014; Arce-Molina et al., 2020; Morris et al., 2020; Sharma and Hasan, 2020; Cho et al., 2021; Hartwig et al., 2021; Wong et al., 2021; Houlihan et al., 2022). Some sensors are substrate-dependent, while some are light-dependent. On the other hand, UAS-mitoGFP is used as a mitochondrial marker to visualize mitochondria in any tissue because, in this case, the mitochondrial import sequence is fused to GFP (Lutas et al., 2012; DeVorkin et al., 2014; Morris et al., 2020; Hartwig et al., 2021; Figure 2A).

## Substrate-dependent sensors

Various substrate-dependent sensors were created to detect substrates like hydrogen peroxide, glutathione, pyruvate, ATP, NAD/NADH, and calcium. To see redox changes in live mitochondria, the UAS-mito-roGFP2-Grx1 is available, which encodes a redox-sensitive GFP cassette fused to glutaredoxin-1 (Grx1) (Albrecht et al., 2014; Krzystek et al., 2021; Houlihan et al., 2022; Figure 2B). The sensor for pyruvate is known as UAS-mito-PyronicSF and consist of a circularly permuted GFP fused to the bacterial pyruvate-sensitive transcription factor PdhR. It binds to pyruvate and induces conformational changes that increase the readout of FRET signal (Arce-Molina et al., 2020). Therefore, this sensor can be used to study mitochondrial metabolism (Figure 2C).

**FIGURE 3:**
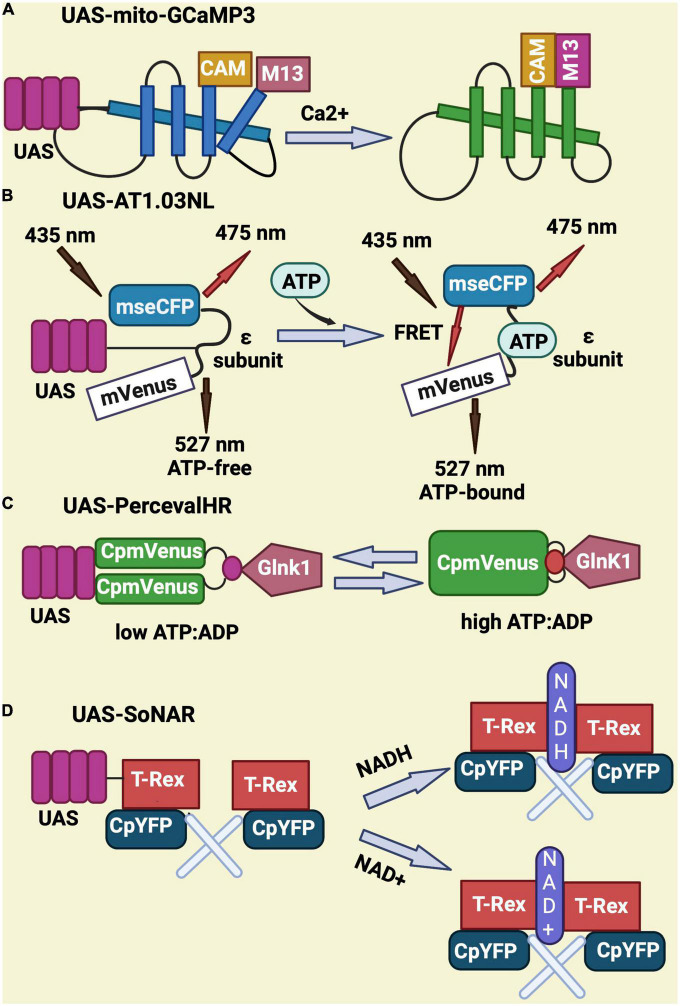
Schematic representation of genetically-encoded sensors for Calcium, ATP, ATP/ADP, NADH, and NAD. **(A)** UAS-mito-GCaMP3 encodes for a circularly permutated EGFP M13 and calmodulin fusion protein that gives GFP signal upon binding to calcium. **(B)** UAS-AT1.03NL consist of two fluorescent proteins (mVenus and mseCFP) and an ATP binding sequence (ε subunit) that elicits a FRET signal upon binding to ATP. **(C)** UAS-PercevalHR contains the ATP-binding protein GlnK1 from *Methanocaldococcus jannaschii* linked to circularly permutated mVenus and allows ATP sensing through conformational changes in Glnk1. **(D)** UAS-SoNar serves as NADH/NAD + sensor and encodes for a fusion of the NADH-binding domain of T-Rex (Rex protein from *Thermus aquaticus*) with a circularly permuted yellow fluorescent protein (cpYFP).

To monitor calcium, the UAS-mito-GCaMP3 was designed to express a circularly permutated EGFP M13/Calmodulin fusion protein under control of the UAS promoter. Upon binding to calcium, this fusion protein undergoes conformational changes that elicit GFP signal (Figure 3A; Lutas et al., 2012; Morris et al., 2020; Sharma and Hasan, 2020). UAS-AT1.03NL is an ATP sensor that consists of two fluorescent proteins (mVenus and mseCFP) linked to the ε subunit from bacterial F0F1-type ATP synthase. The ε subunit has two C-terminal helices and an N-terminal barrel domain. Low FRET efficiency results from the loose and flexible subunit separating two fluorescent proteins in ATP-free mode. When ATP is bound, the ε subunit configuration switches from open to close, bringing the two fluorescent proteins closer together and increasing FRET efficiency (Imamura et al., 2009; Tsuyama et al., 2013; Dong and Zhao, 2016; Cho et al., 2021; Figure 3B). UAS-PercevalHR is also used as ATP/ADP sensor and contains the ATP-binding protein GlnK1 from *Methanocaldococcus jannaschii* and circularly permutated monomeric Venus (CpmVenus) connected by a peptide linker. Upon binding to ATP, the T-loop of GlnK1 undergoes a dramatic conformational change from a loose, disordered structure to a tight, ordered loop, which integrates CpmVenus into the T-loop for sensing ATP (Berg et al., 2009). It has an excitation peak at 405 nm for ATP binding and 488 nm for ADP binding (Figure 3C; Broyles et al., 2018; Morris et al., 2020; Wong et al., 2021). Lastly, the UAS-SoNaR transgene is used as NADH/NAD + sensor. It has the NADH-binding domain of Rex protein from *Thermus aquaticus* (T-Rex) connected to a circularly permuted yellow fluorescent protein (cpYFP) (Figure 3D). It has excitation at 420 or 485 nm with emission at 528 nm to determine NADH/NAD + ratio (Zhao et al., 2015; Bonnay et al., 2020; Morris et al., 2020).

## Light-dependent sensors

**FIGURE 4:**
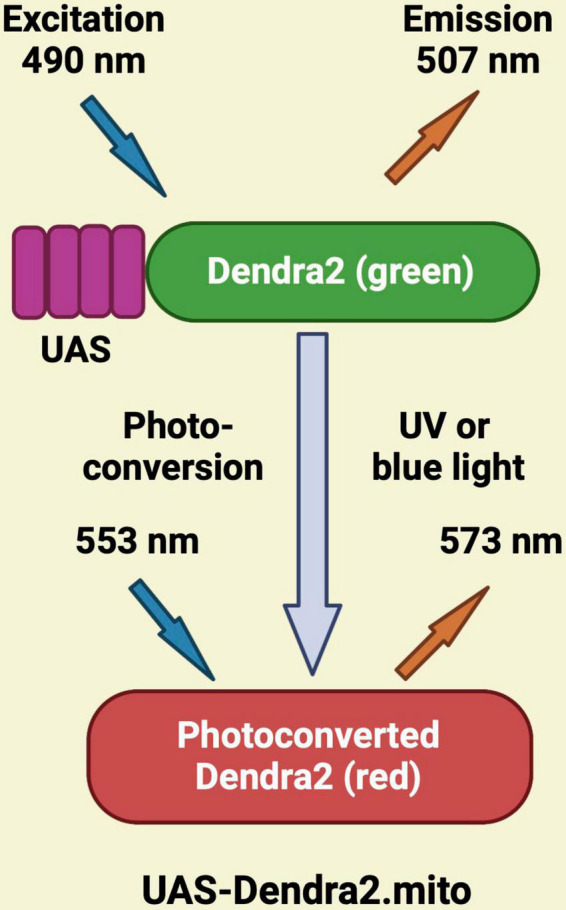
Schematic representation of the photo-convertible process associated with UAS-Dendra2.mito. Green Dendra2 is photo-converted to the red form upon UV or blue light exposure.

UAS-Dendra2.mito is a light-dependent sensor where the photoconvertible protein Dendra2 (green) from the filamentous fungus *A. nidulans* is tagged to the mitochondrial matrix. The green fluorescence of this tagged protein can be localized and examined prior to irradiation. The region of interest within a cell or the entire cell is then exposed to UV (405 nm) or blue light (488 nm) lasers. Dendra2 is immediately photo-converted from green to red fluorescence by UV or blue light (Figure 4). It is used to monitor mitochondria over time (Hwang et al., 2014; Perez-de-Nanclares-Arregi and Etxebeste, 2014; Bertolin et al., 2018). This sensor allows easy tracking of green signal before photoconversion and the corresponding shift to red fluorescence upon UV or blue light irradiation. Importantly, with excitation and emission occurring at 553 and 573 nm, respectively, the activated red Dendra2 signal exhibits high photo-stability (Perez-de-Nanclares-Arregi and Etxebeste, 2014).

## Mitochondrial dysfunction in Aβ-expressing flies

Wang and Davis (2021) recently assessed mitochondrial function and dynamics in flies expressing Aβ in the mushroom body neurons through super-resolution microscopy, calcium imaging and behavioral assays. They found that Aβ induces mitochondrial fragmentation and dysfunction at a very early age, consistent with detectable apoptosis. Interestingly, learning was impaired much later than the initial mitochondrial abnormalities, confirming the proximal role of mitochondria in AD pathogenesis. In terms of structural changes, Aβ was found to induce formation of aberrant mitochondria with a build-up of vacuoles or damaged cristae in the pre-synapse of the fly dorsal longitudinal flight muscle (DLM). In this case, Aβ decreased the age-dependent anterograde and retrograde axonal trafficking of mitochondria (Zhao et al., 2010). To determine if manipulation of mitochondrial fission could modify Aβ-induced phenotypes, the fission regulator Drp1 was pan-neuronally co-expressed with Aβ. This work demonstrated that overexpression of Drp1 improves survival, climbing capacity, neuronal degeneration and ATP levels in Aβ flies (Lv et al., 2017). On the other hand, over-expression of Aβ in all neurons decreases *drp1* and m*arf* mRNA levels in older flies (Abtahi et al., 2020).

Another protein essential for mitochondrial function and transport is Milton. It connects Miro to kinesin, (Stowers et al., 2002) and enables them to move in axons and dendrites (Glater et al., 2006). Knock-down of Milton enhanced Aβ-induced locomotion defects. Consistently, heterozygous *miro* mutants led to an enhancement of Aβ-induced locomotor impairment associated with mitochondrial mislocalization (Iijima-Ando et al., 2009), whereas over-expression of Miro improved the eye phenotype, climbing performance and ATP levels in Aβ flies (Panchal and Tiwari, 2020).

To understand the effects of Aβ on mitochondrial distribution, Aβ was expressed in glutamatergic motor neurons of the fly leg. Aβ significantly reduces the number of mitochondria in the motor neurons and shortens the fly lifespan when overexpressed in glutamatergic neurons (Fernius et al., 2017). This suggests that Aβ affects mitochondrial distribution in the neuron, possibly contributing to the reduction in life span.

Mitochondria and endoplasmic reticulum connect to create mitochondria-ER contact sites (MERCs), which facilitates the exchange of lipids and calcium ions. However, MERCs dysfunction or miscommunication affects ATP generation and mitochondrial division by disturbing calcium shuttling and possibly through interaction with Drp1 and mitochondrial fission factor (MFF), although the molecular mechanism is not well understood (Rowland and Voeltz, 2012; Wilson and Metzakopian, 2021). This is relevant because alterations of the mitochondria–endoplasmic reticulum contacts have been reported in AD (Schon and Area-Gomez, 2013). In an effort to improve contacts between mitochondria and ER, synthetic linkers have been designed to enhance the proximity between both organelles. For instance, over-expression of a synthetic linker carrying mitochondrial and ER targeting sequences extended lifespan and suppressed climbing deficits in Aβ flies, which suggests that improving the interaction between mitochondria and ER could alleviate AD pathologies associated with mitochondrial dysfunction (Garrido-Maraver et al., 2020). However, a different team recently demonstrated that the knockdown of *pdzd8*, a putative *Drosophila* homolog of the mammalian MERC tethering protein, decreases contacts between the ER and mitochondria and restores locomotor deficits in Aβ flies (Hewitt et al., 2022). Thus, further research is needed to clarify these contradictory findings.

Calcium homeostasis is very important for proper mitochondrial function and must be preserved. Flies expressing human Aβ in the mushroom body have significantly reduced calcium import compared to control flies, suggesting an Aβ-mediated impairment of mitochondrial function in mushroom body neurons (Wang and Davis, 2021). In addition, pan-neuronal expression of Aβ reduced the levels of phosphoproteins predicted to be substrates of PKA, indicating that Aβ42 might modulate cAMP/PKA signaling (Iijima-Ando et al., 2009). This cAMP/PKA signaling is known to be affected by mitochondrial dysfunction which, in turn, reduces synaptic strength and prevents synaptic vesicle movement in the presynaptic terminal (Verstreken et al., 2005).

NDUFS3, a core component of the mitochondrial complex, is involved in the electron transport chain. Its expression is down-regulated in Aβ-expressing flies, which causes a decrease in the generation of ATP (Lin et al., 2021). Given that vitamin K serves as a mitochondrial electron transporter during oxidative respiration (Vos et al., 2012), there is an interest in investigating its therapeutic potential in fly models of AD. For instance, Lin et al. (2021) treated Aβ flies with vitamin K, which led to an improvement of mitochondrial function, reduction of Aβ neurotoxicity, and autophagy activation with concomitant increase in NDUFS3 expression and ATP levels.

**FIGURE 5:**
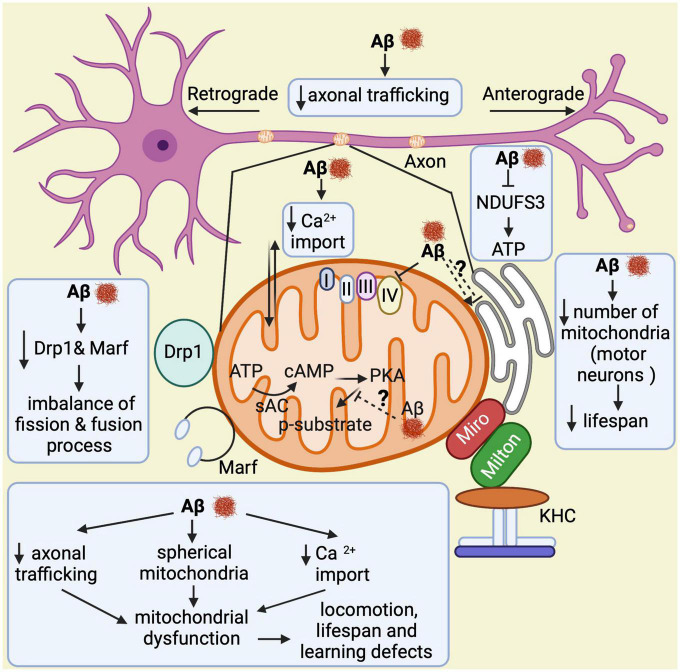
Overview of mitochondrial abnormalities found in Aβ-expressing flies. Aβ induces formation of spherical mitochondria, which disrupts their function. It also decreases calcium import into the mitochondria and affects their axonal trafficking, which impacts learning, lifespan, and locomotor behaviors. Aβ down-regulates NDUFS3, a crucial factor for the electron transport chain, resulting in low ATP production. In motor neurons, it reduces the number of mitochondria leading to shortened lifespan. Furthermore, Aβ decreases Drp1 and Marf expression, which causes an imbalance in the fission and fusion processes. Lastly, it seems to alter the mitochondria–endoplasmic reticulum contacts, although contradictory results have been found in this regard. Thus, this is highlighted with a question mark.

Taken together, all these findings demonstrate that Aβ over-expression in flies triggers abnormal structural and functional changes in mitochondria, disrupts their dynamics and transport, and impairs learning and memory in late stages of the disease, all of which is relevant to understand AD pathogenesis (Figure 5).

## Mitochondrial dysfunction in tau-expressing flies

Tau is a protein involved in the polymerization and stabilization of microtubules and is also associated with the axonal transport of sub-cellular organelles (Gendron and Petrucelli, 2009; Dolan and Johnson, 2010). Phosphorylation of tau reduces its binding affinity for the tubulin subunits of microtubules, which enhances the self-aggregation and fibrillization of phosphorylated tau (Cohen et al., 2011; Cisek et al., 2014; Singh et al., 2015) and leads to defects in axonal transport of mitochondria (Ittner and Götz, 2011; Mondragón-Rodríguez et al., 2013; Mietelska-Porowska et al., 2014). Over-expression of human wild-type and mutant tau R406W in flies induces elongation of mitochondria, resulting in mitochondrial dysfunction and apoptotic neurodegeneration with cell cycle activation (Wittmann et al., 2001; Khurana et al., 2006). Tau-dependent activation of the cell cycle requires tau phosphorylation and it has been shown that TOR signaling is also involved in the cell-cycle activation that mediates tau-induced neurodegeneration (Khurana et al., 2006). Interestingly, stimulation of mitochondrial fission by concomitantly increasing the expression of *Drp1* and reducing *Marf* levels reversed mitochondrial elongation and alleviated tau neurotoxicity in flies, suggesting that restoring the proper balance of mitochondrial fission and fusion is necessary to alleviate mitochondrial dysfunction and cell cycle-mediated cell death (DuBoff et al., 2012). In contrast, increasing fusion by upregulation of *Marf* and downregulation of *Drp1* further increased the mitochondrial length in tau flies, resulting in more aggressive neurodegeneration (DuBoff et al., 2012).

Dynamin-related-protein1 is a cytoplasmic protein that translocates to the mitochondrial outer membrane to drive mitochondrial fission; however, in the context of mutant tau overexpression, Drp1 staining does not colocalize with mitoGFP and stays primarily in the cytosol, as evidenced by its distribution in cytoplasmic and mitochondrial fractions from fly heads (Frank et al., 2001). This tau-related blockage of Drp1 translocation is thought to be mediated by stabilization of actin because reversing actin stabilization rescues tau-induced mitochondrial defects (DuBoff et al., 2012). Another study demonstrated that over-expression of leucine-rich repeat kinase 2 (LRRK2) also increases tau neurotoxicity through excessive actin stabilization and subsequent mislocalization of Drp1 (Bardai et al., 2018). This seems to be a relevant pathway as pharmacological suppression of actin polymerization was found to reverse neurodegeneration and mitochondrial impairments in tau transgenic flies (Bardai et al., 2018). On the other hand, given that the balance between fusion and fission is disturbed in AD (Wang et al., 2008a), Abtahi and coworkers looked at how tau affects the expression of *Marf* and *Drp1*, which are essential for mitochondrial fusion and fission, respectively, (Abtahi et al., 2020). The authors found that pan-neural expression of both wild-type and mutant tau R406W in flies decreased the expression of *Marf* mRNA in older flies, suggesting that the decline in the mitochondrial fusion process takes place at a later stages in AD. The expression of *Drp1* is modulated differently by wild-type and mutant tau; wild-type tau up-regulates *Drp1* mRNA, whereas mutant tau down-regulates it. Despite the fact that mutant and wild type tau express *Marf* and *Drp1* differently, the increased ratio of *Drp1*/*Marf* suggests that both wild type and mutant tau display more mitochondrial fission (Abtahi et al., 2020), uncovering an imbalance between fusion and fission in tau flies.

In *Drosophila* larval motor neurons, overexpression of human tau (0N3R) disrupts axonal transport as well as the morphology and function of neuromuscular junctions (Chee et al., 2005). This is caused by a marked decrease in the amount of detectable mitochondria in the pre-synaptic terminal, which causes synaptic dysfunction accompanied by a lower number of functional mitochondria (Chee et al., 2005). To understand the effects of tau on mitochondrial distribution, both UAS-tau^0N4R^ and UAS-tau^0N4R–E14^ were expressed in the fly leg neurons. The results showed that “clump-like” aggregation of mitochondria is seen in the motor neurons projecting into the muscles while the distribution of mitochondria is even in control flies, indicating that tau affects mitochondrial distribution (Fernius et al., 2017). Accordingly, wild-type UAS-tau^0N4R^ and UAS-tau^0N4R–E14^ expression in glutamatergic neurons significantly shorten the fly lifespan (Fernius et al., 2017).

A recent genome-wide RNAi screen in flies expressing human mutant tau pan-neuronally led to the identification of several modifiers involved in the mitochondrial pathway, such as biotinidase, *NDUFS4*, *ALDH6A1*, and *TFB1M* (Lohr et al., 2020). The authors found that the knock-down of biotinidase in tau flies disrupts the structure and function of mitochondria, the function of carboxylase enzymes, and leads to a more aggressive neurodegeneration. Interestingly, administration of biotin through feeding rescues toxicity of both wild-type and mutant tau in transgenic flies (Lohr et al., 2020). This is relevant because the authors also found reduced carboxylase biotinylation in the brain of some AD patients. However, the extent to which biotin levels contribute to AD pathogenesis is largely unknown. On the other hand, to understand the possible interaction between toxicity of tau and axonal mitochondria, the adaptor proteins essential for axonal mitochondrial transport, Milton and Miro, were knocked-down in flies expressing wild type tau. Knock-down of either Milton or Miro enhances tau-induced neurodegeneration. Moreover, Milton knock-down accelerates the accumulation of autophagic bodies and vacuole formation in presynaptic vesicles and axons (Iijima-Ando et al., 2012). Additionally, it increases tau phosphorylation at Ser262 via the partitioning defective-1 (PAR-1) protein, which decreases tau ability to bind microtubules. This suggests that both tau phosphorylation at Ser262 and PAR-1 are essential for enhancing tau-induced axon degeneration in Milton knockdown. Whereas pan-neuronal knockdown of Milton or Miro results in age-dependent neurodegeneration in the fly brain, knock-down of PAR-1 or endogenous fly tau suppresses Milton knockdown-induced neurodegeneration, demonstrating that both PAR-1 and tau participate in Milton knockdown-mediated neuropathology (Iijima-Ando et al., 2012).

**FIGURE 6:**
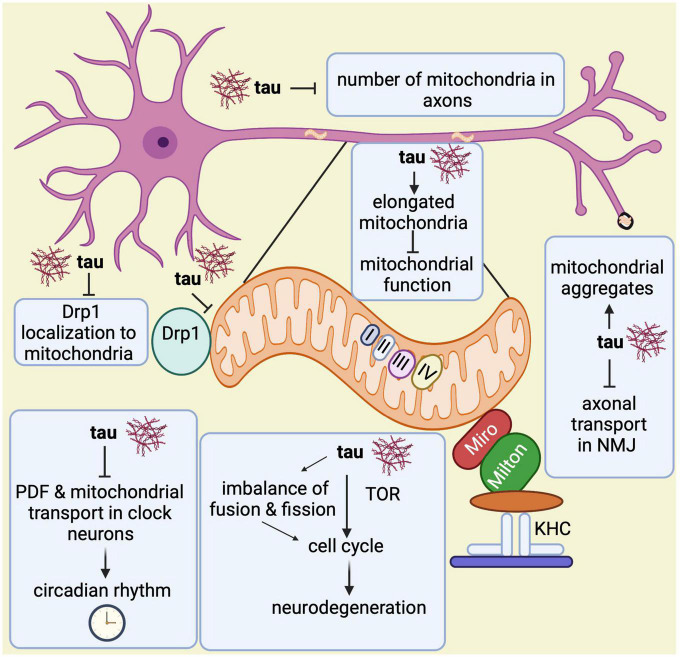
Overview of mitochondrial abnormalities found in tau-expressing flies. Tau induces elongated mitochondria, which causes mitochondrial dysfunction and apoptotic neurodegeneration with cell cycle activation. The cell cycle activation brings neurodegeneration via TOR and through an imbalance in the fusion and fission processes. Tau disrupts the circadian rhythm by inhibiting the neuropeptide pigment dispersing factor (PDF) and reducing mitochondrial transport in axons of clock neurons. Additionally, it prevents Drp1 from localizing to the outer mitochondrial membrane, which affects the fission process. Tau also disrupts axonal transport in the neuromuscular junction (NMJ) leading to mitochondrial aggregation. Lastly, it has been found that tau also reduces mitochondrial numbers in axons.

Lastly, another study showed that pan-neuronal expression of wild-type and phosphomimetic mutant tau (tauE14) disrupts the circadian rhythm (Zhang et al., 2022). While wild type tau expression in clock neurons reduces the levels of neuropeptide pigment dispersing factor (PDF), a neurotransmitter essential for circadian function in *Drosophila*, expression of tauE14 in clock neurons disrupts the circadian rhythm and reduces PDF distribution in the dorsal axonal projections. Interestingly, tauE14 also induces a complete loss of mitochondria in dorsal projections indicating that tauE14 impairs axonal transport of neuropeptides and mitochondria in circadian pacemaker neurons, affecting circadian rhythm (Zhang et al., 2022). Further studies will be required to better understand the association between circadian rhythm, mitochondrial biology and Alzheimer’s disease. In summary, all these observations highlight multiple ways in which abnormal tau contributes to mitochondrial malfunction (Figure 6).

## Concluding remarks and future directions

Alzheimer’s disease is a devastating neurodegenerative brain disorder characterized by extracellular Aβ plaques and intracellular aggregates of hyperphosphorylated tau, along with progressive cognitive decline. Despite decades of research and impressive efforts from multiple groups, there is still no treatment available for this dreadful disorder. Recent evidence suggested that AD and other neurodegenerative diseases are impacted by mitochondrial dysfunction, an area that may provide new targets for future therapeutic strategies.

In this review, we emphasized how *Drosophila* closely resembles molecular and pathological features of Aβ- and tau-related mitochondrial disfunction observed in AD. We compiled relevant studies in flies showing how Aβ42 or tau affect the structure of mitochondria, mitochondrial dynamics, calcium homeostasis, axonal transport of mitochondria, cAMP/PKA signaling, mitochondria-ER contact sites as well as the expression of several mitochondrial factors. We also discussed available assays and tools for examining mitochondrial function and dynamics in *Drosophila*. Considering the flexibility and power of *Drosophila* genetics, it is clear that this model organism will continue improving our understanding of the critical role of mitochondria in AD pathogenesis and its crosstalk with other pathomechanisms.

It is worth noting that there are still many opportunities for *Drosophila*-based research in this field. For instance a recent study found that the glutathione S-transferase (GST) Gfzf prevents mitochondrial hyperfusion in axons and regulates mitochondrial dynamics (Smith et al., 2019). This is important because several GST polymorphisms in humans have been associated with the development of AD (Allen et al., 2012). Thus, future manipulation of Gfzf in fly models of AD will help understand the potential contribution of GST activity to this devastating disorder.

One limitation of the studies discussed here is that they were performed in either Aβ42- or tau-expressing flies. Since Aβ42 and tau display synergistic interactions (Zhang H. et al., 2021), it will be imperative to study mitochondrial dynamics and function in flies co-expressing Aβ42 and tau to provide a more physiological context. This is because a recent longitudinal positron emission tomography (PET) study found that Aβ accelerated tau deposition in the inferior temporal cortex of older people with cognitively normal function over a 7-year follow-up period and that the rate of such accumulation was linked to the degree of cognitive decline (Hanseeuw et al., 2019). This finding is consistent with another PET study in cognitively healthy individuals showing that Aβ-tau interactions (rather than Aβ or tau alone) accelerated cognitive decline (Sperling et al., 2019). Moreover, individuals with primary age-related tauopathy, who exhibited equivalent tau burdens but negligible amounts of Aβ, had lesser high-molecular-weight (HMW) tau levels than patients with AD, who showed typical Aβ-plaque and tau-tangle burden (Bennett et al., 2017). Taken together, these studies confirm the synergistic interaction between Aβ and tau in AD pathology and emphasize the need of using fly models with concurrent Aβ and tau pathologies. On the other hand, TDP-43, an RNA/DNA binding protein linked to frontotemporal lobar degeneration and amyotrophic lateral sclerosis, was recently found to mediate prominent structural and functional damage to mitochondria along with activation of the mitochondrial unfolded protein response (UPR^mt^) (Wang et al., 2019). This is also relevant because more than 50% of AD cases display TDP-43 pathology in the brain (Meneses et al., 2021). It is, therefore, critical to concurrently manipulate Aβ42, tau and TDP-43 in transgenic flies to decipher their overall mitochondrial insults in AD cases with TDP-43 pathology. Approaching these and other unknown aspects of mitochondrial dysfunction with *Drosophila* will provide a more comprehensive portrait of molecular abnormalities and, thus, may lead to the identification of multiple and promising therapeutic targets in the years to come.

## Author contributions

VV and DER-L conceived the original idea and revised the manuscript. VV and JM designed the outlines of the study, performed the literature review, wrote the first draft, and prepared the figures. All authors read and approved the final manuscript.
